# Diabetic Nephropathy and Its Risk Factors in a Society with a Type 2 Diabetes Epidemic: A Saudi National Diabetes Registry-Based Study

**DOI:** 10.1371/journal.pone.0088956

**Published:** 2014-02-21

**Authors:** Khalid Al-Rubeaan, Amira M. Youssef, Shazia N. Subhani, Najlaa A. Ahmad, Ahmad H. Al-Sharqawi, Hind M. Al-Mutlaq, Satish K. David, Dhekra AlNaqeb

**Affiliations:** 1 University Diabetes Center, College of Medicine, King Saud University, Riyadh, Saudi Arabia; 2 Registry Department, University Diabetes Center, King Saud University, Riyadh, Saudi Arabia; 3 Department of Biostatistics, Epidemiology and Scientific Computing, King Faisal Specialist Hospital and Research Centre, Riyadh, Saudi Arabia; 4 Biostatistics Department, University Diabetes Center, King Saud University, Riyadh, Saudi Arabia; 5 Family and Community Medicine Department, Qassim University, Qassim, Saudi Arabia; 6 Information Technology Department, Strategic Center for Diabetes Research, King Saud University, Riyadh, Saudi Arabia; 7 Research Department, University Diabetes Center, King Saud University, Riyadh Saudi Arabia; TGen, United States of America

## Abstract

**Aims:**

The prevalence of diabetic nephropathy and its risk factors have not been studied in a society known to have diabetes epidemic like Saudi Arabia. Using a large data base registry will provide a better understanding and accurate assessment of this chronic complication and its related risk factors.

**Methodology:**

A total of 54,670 patients with type 2 diabetes aged ≥25 years were selected from the Saudi National Diabetes Registry (SNDR) and analyzed for the presence of diabetic nephropathy. The American Diabetes Association (ADA) criterion was used to identify cases with microalbuminuria, macroalbuminuria and end stage renal disease (ESRD) for prevalence estimation and risk factor assessment.

**Results:**

The overall prevalence of diabetic nephropathy was 10.8%, divided into 1.2% microalbuminuria, 8.1%macroalbuninuria and 1.5% ESRD. Age and diabetes duration as important risk factors have a strong impact on the prevalence of diabetic nephropathy, ranging from 3.7% in patients aged 25–44 years and a duration of >5 years, to 21.8% in patients ≥65 years with a diabetes duration of ≥15 years. Diabetes duration, retinopathy, neuropathy, hypertension, age >45 years, hyperlipidemia, male gender, smoking, and chronologically, poor glycemic control has a significantly high risk for diabetic nephropathy.

**Conclusion:**

The prevalence of diabetic nephropathy is underestimated as a result of a shortage of screening programs. Risk factors related to diabetic nephropathy in this society are similar to other societies. There is thus an urgent need for screening and prevention programs for diabetic nephropathy among the Saudi population.

## Introduction

Electronic medical health systems have made chronic diseases like diabetes mellitus easier to monitor and understand through large data base registries. Diabetes Registry is gaining popularity nowadays as a result of its advantages in collecting and characterizing data about the disease for clinical and scientific studies. The prevalence of diabetes is increasing globally as a result of urbanization, human aging and lifestyle changes. Diabetes registries track this disease behavior, and provide a better understanding of its clinical, social, cultural and economic impact. Disease registries not only improve perception of the disease, but help in the health planning and assessment of health care quality. They are considered to be a reliable source of epidemiology data that serve to highlight both morbidity and mortality from such diseases [1]. Many countries have adopted renal registries, such as Finland, Hong Kong and the United Kingdom, and this shows that diabetic renal disease remain the single most common cause of renal failure, amounting to 24.8% [2]. Health care stakeholders are currently more interested than before in data provided by registries, and this may explain the large increase in the number of registries at a global level [3].

Diabetes prevalence in the Middle Eastern region is considered to be the highest, averaging 10.9%. In 2011, six Middle Eastern countries were listed among the top ten countries with a high prevalence of diabetes for ages 20–79 years, with Saudi Arabia ranking seventh [4]. Abnormal glucose metabolism has reached an epidemic stage in the Kingdom, with diabetes and impaired fasting glucose crude prevalence being 23.7% and 14.1% respectively, accounting for 37.8% of Saudis aged between 30 to 70 years [5].

Evidence based medicine shows that diabetes morbidity and mortality are attributed to its chronic complications. According to ADA (2012), Diabetic nephropathy (DN) is a microvascular complication known to be the leading cause of ESRD worldwide, and is associated with increased cardiovascular risk. Epidemiology studies of type 2 diabetic patients show that DN prevalence ranges from 7.6% to 55% [6], while in different international registries it varies between 11.5% in United Kingdom and 42.9% in Thailand [7], [8]. This large variation in DN prevalence reported by registries may be related to registry size, screening and management practice, and can be improved with larger registries, and by implementing standardized practice, especially a longer follow up period.

Saudi Arabia, known as a society with high type 2 diabetes prevalence, has limited data concerning diabetic nephropathy can be used for better understanding of this chronic complication. A study in 2008 unexpectedly considered DN to be the most prevalent chronic complication among Saudi type 2 diabetic patients, accounting for 32.1%, which could be attributed to the sample selection methodology used [9]. Hypertension, longer diabetes duration, poor glycemic control, dyslipedemia, smoking, obesity, male gender and presence of retinopathy are well known risk factors for DN in type 1 diabetes [10]. These risk factors were not tested on a large number of diabetic patients in a high diabetes prevalent society so as to validate the importance of such risk factors. Hazard ratio tested for the progression of diabetic nephropathy among Saudis showed significant values for presence of retinopathy, male gender, diabetes duration >10 years and presence of hypertension [11].

## Methodology

SNDR, hosting more than 100.000 Saudi diabetic patients, can be the best environment to study DN and its risk factors. It is a specially designed electronic web-based data system which incorporates demographic data, diabetic and social history with clinical and biochemical parameters. This registry includes the presence of chronic complications, namely neuropathy, retinopathy, nephropathy, and vasculopathy, as well as associated diseases like hypertension, hyperlipidemia and thyroid disease. The design and development of a web-based SNDR has already been explained in a previously published paper, available at http://www.jmir.org/2013/9/e202, and the registry website can be accessed from http://www.diabetes.org.sa. This diabetes national registry is one of the Saudi strategic research projects approved and funded by King Abdulaziz city for science and Technology (KACST), which is the official largest funding body in the kingdom.

This is a cross-sectional, randomized observational study, where 67,075 Saudi diabetic patients (51.2% males and 48.8% females) were selected from SNDR to assess the presence of diabetic nephropathy after been de-identified. ADA criteria were used to identify diabetes types and DN diagnostic criteria. The selected sample was categorized according to DN classification into microalbuminuria, macroalbuminuria, and ESRD. Nephropathy was excluded if a patient had no proteinuria or albumin excretion <30 µg\mg creatinine and normal glomerular filtration rate (GFR) ≥90 ml\min per 1.73 m^2^ body surface area. Microalbuminuria is diagnosed when albumin is between 30–299 µg\mg creatinine, and macroalbuminuria when albumin excretion ≥300 µg\mg creatinine. Patients were identified with ESRD if they had GFR <30 ml\min per 1.73 m^2^ body surface area, or had been diagnosed by their treating physician [8]. Patients on dialysis at the time of the registry, or who had received kidney transplant, were considered to have ESRD.

**Figure 1:**
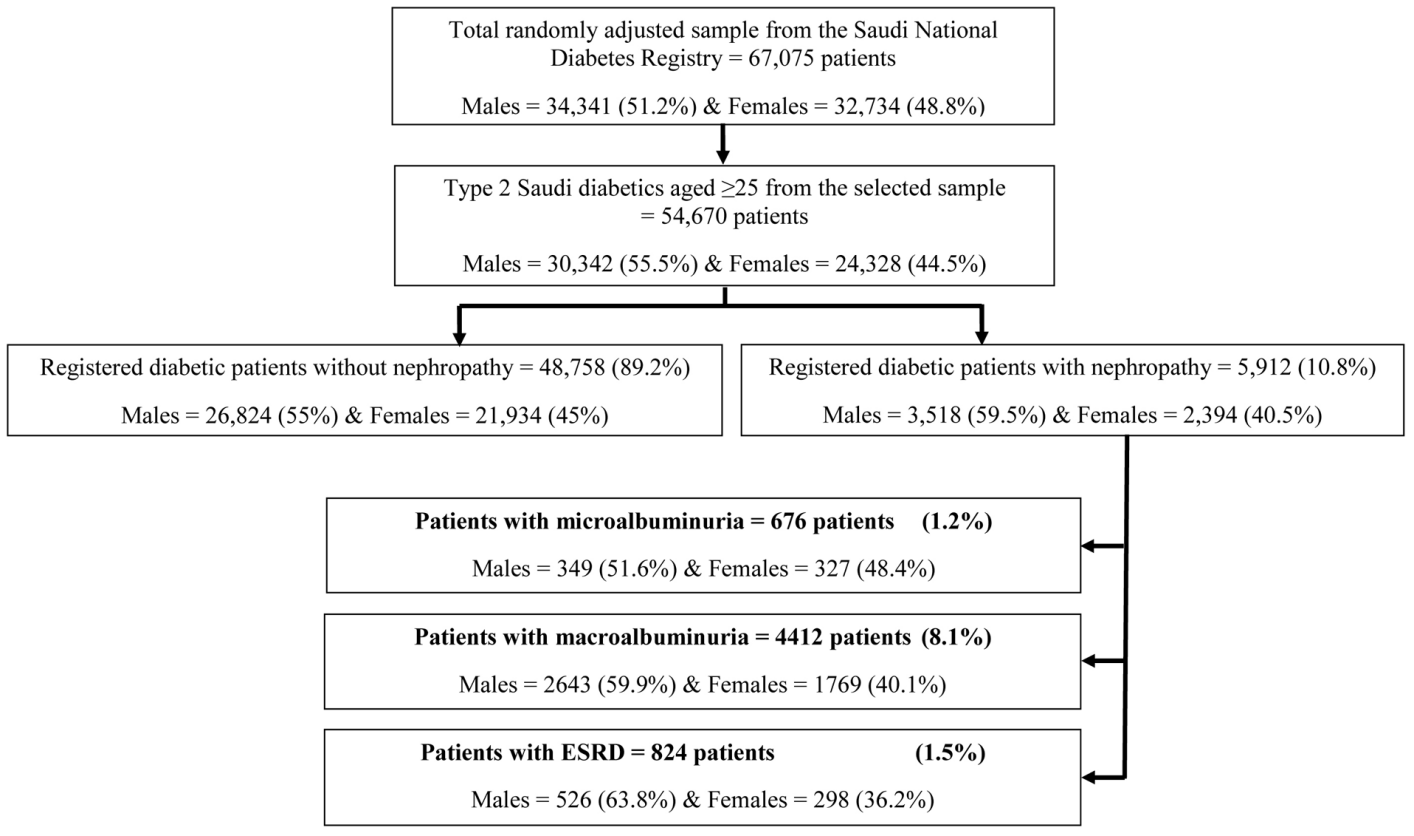
Sampling methodology for patients with diabetic nephropathy and its different types, among type 2 diabetes patients aged ≥25 years from the Saudi National Diabetes registry.

Figure 1 shows the selection of type 2 Saudi diabetic patients aged ≥25 years totaling at 54,670 patients, of which 30,342 (55.5%) were males and 24,328 (44.5%) females. Diabetic nephropathy was found in 5,912 (10.8%), of which 59.5% were males and 40.5% females. Microalbuminuria was found in 676 (1.2%) patients, of which 51.6% were males and 48.4% were females. Macroalbuminuria prevalence was 8.1%, of which 59.9% were males and 40.1% were females. ESRD affected 1.5% of the total sample, with 63.8% being males and 36.2% being females.

### Statistical Analysis

All data were entered into the centralized database via the web application SNDR, and was analyzed using SPSS program version 17.0. Descriptive analyses and frequency tables were performed using this program for all variables. Chi square test (χ^2^) was used for categorical variables such as gender and smoking status, while t-test was used for continuous variables such as age, duration of diabetes and body measurements, including height, weight, Body Mass Index (BMI), and HbA1c. Odds ratio (with 95% confidence interval) and p-value of 0.05 or less were used as a level of significance for assessing the risk factors of diabetic nephropathy. The risk factors of DN were plotted using Graph Pad software.

## Results

A total of 5,912 patients fulfilled the diabetic nephropathy criteria from the selected sample, accounting for 10.8% of the 54.670 Saudi type 2 diabetic aged ≥25 years patients, divided into Microalbuminuria, macroalbuminuria and ESRD, representing 11%, 75% and 14% of the total nephropathic patients respectively.

Table 1 shows the descriptive analysis of the selected sample and nephropathy cases. The total sample mean age was 59.91 (±12.72) years, and BMI is 30.46 (±6.30) kg/m^2^ with mean weight and height being 78.55 (±16.50) kg and 160.24 (±9.74) cm respectively. Mean diabetes duration was 13.55 (±8.14) years.

**Table 1:** Descriptive analysis of the selected cohort according to the Nephropathy type.

Variables	Selected sample	No Nephropathy	Diabetic Nephropathy							
			Classification according to diabetic nephropathy status							
	Total (54670)	Total (48758)	Total (5912)		Microalbuminuria (676)		Macroalbuminuria (4412)		ESRD (824)	
	Mean(SD)	Mean(SD)	Mean(SD)	Pvalue*	Mean(SD)	Pvalue*	Mean(SD)	Pvalue*	Mean(SD)	Pvalue*
**Age (years)**	59.91(±12.72)	59.41(±12.71)	63.99(±12.06)	<0.0001	57.83(±11.58)	0.014	64.34(±11.87)	<0.0001	64.29(±11.12)	<0.0001
**Weight (kg)**	78.55(±16.50)	78.73(±16.52)	77.20(±16.27)	<0.0001	80.31(±14.81)	0.044	76.96(±16.57)	<0.0001	71.82(±15.70)	<0.0001
**Height (cm)**	160.24(±9.74)	160.29(±9.73)	159.93(±9.77)	0.02	160.66(±9.74)	0.485	159.71(±9.20)	0.006	160.86(±10.62)	0.381
**BMI (kg/m^2^)**	30.46(±6.30)	30.52(±6.29)	30.07(±6.38)	<0.0001	31.06(±6.11)	0.11	30.11(±6.44)	0.004	27.70(±6.54)	<0.0001
**DM duration (years)**	13.55(±8.14)	12.85(±7.81)	18.81(±8.61)	<0.0001	13.47(±7.77)	0.128	19.40(±8.32)	<0.0001	19.59(±8.26)	<0.0001

*P value is the difference between non-nephropathic patients and microabluminureia, macroalbuminuria, and ESRD patients. Frequency analysis was adjusted according to the data availability.

When comparing patients with and without nephropathy, nephropathic patients were significantly older (63.99 vs 59.41 years) and had longer diabetes duration (18.81 vs. 12.85 years), (p<0.0001). Non-nephropathic patients had significantly higher weight, height and BMI than total nephropathic, (p<0.0001, 0.02, <0.001) respectively and macralbuminuria patients, (p = 0.006). Microalbuminuria patients had a significantly higher weight than non-nephropathic. Patients with ESRD were significantly lower in weight and BMI when compared with non nephropathic. Mean age was significantly higher in microalbuminuria, macroalbuminuria and ESRD patients, representing 57.83(±11.58), 64.34(±11.87) and 64.29(±11.12) years, (p = 0.014, <0.0001,<0.0001) respectively. Diabetes duration was significantly longer in patients with macroalbuminuria and ESRD, but not for patients with microalbuminuria, at 19.40, 19.59 and 13.47 years respectively, (p<0.0001, <0.0001, 0.128).

Table 2 shows the descriptive analysis of the selected sample and nephropathy cases.

**Table 2:** Frequency analysis of the selected cohort according to the Nephropathy type.

Variables		Selected sample	No Nephropathy	Diabetic Nephropathy							
				Classification according to diabetic nephropathy status							
		Total (54670)	Total (48758)	Total (5912)		Microalbuminuria (676)		Macroalbuminuria (4412)		ESRD (824)	
Category	subcategory	Number (%)	Number (%)	Number (%)	P value*	Number (%)	P value*	Number (%)	P value*	Number (%)	P value*
**Age**	**25–44 years**	5765 (10.5%)	5440 (11.2%)	325 (5.5%)	<0.0001	49 (12.3%)	0.455	123 (4.7%)	<0.0001	21 (4.3%)	<0.0001
	**45–64 years**	28548 (52.2%)	25988 (53.3%)	2560 (43.3%)	<0.0001	238 (59.9%)	0.008	1088 (42%)	<0.0001	210 (43.4%)	<0.0001
	**≥65 years**	20357 (37.2%)	17330 (35.5%)	3027 (51.2%)	<0.0001	110 (27.7%)	<0.0001	1379 (53.2%)	<0.0001	253 (52.3%)	0.001
**Gender**	**Male**	30342 (55.5%)	26824 (55%)	3518 (59.5%)	<0.0001	206 (51.9%)	0.213	1559 (60.2%)	<0.0001	311 (64.3%)	<0.0001
	**Female**	24328 (44.5%)	21934 (45%)	2394 (40.5%)	<0.0001	191 (48.1%)	0.213	1031 (39.8%)	<0.0001	173 (35.7%)	<0.0001
**Marital Status**	**Single**	1069 (2%)	985 (2.1%)	84 (1.5%)	0.001	5 (1.3%)	0.284	30 (1.2%)	0.002	15 (3.2%)	0.084
	**Married**	48755 (91.6%)	43505 (91.6%)	5250 (90.9%)	0.053	352 (91.2%)	0.75	2289 (89.8%)	0.001	421 (90.5%)	0.392
	**Divorced**	600 (1.1%)	532(1.1%)	68 (1.2%)	0.7	5 (1.3%)	0.746	38 (1.5%)	0.086	3 (0.6%)	0.331
	**Widow**	2825 (5.3%)	2451 (5.2%)	374 (6.5%)	<0.0001	24 (6.2%)	0.351	191 (7.5%)	<0.0001	26 (5.6%)	0.678
**Family history of DM**	**Yes**	14008 (36.8%)	11975 (35.8%)	2033 (43.9%)	<0.0001	160 (49.4%)	<0.0001	1027 (45.1%)	<0.0001	113 (36.5%)	0.866
**Smoking**	**Yes**	4366 (10.6%)	3796 (10.4%)	647 (10.9%)	0.001	64 (9.5%)	0.645	468 (10.6%)	0.734	115 (13.9%)	0.029
**BMI**	**≤25 kg/m^2^**	6075 (18%)	5184 (17.7%)	891 (20.4%)	<0.0001	46 (13.8%)	0.067	441 (20%)	0.006	86 (35.7%)	<0.0001
	**25–29.9 kg/m^2^**	11399 (33.8%)	9913 (33.7%)	1486 (34%)	0.721	106 (31.8%)	0.457	752 (34.1%)	0.782	77 (32%)	0.552
	**≥30 kg/m^2^**	16246 (48.2%)	14258 (48.6%)	1988 (45.5%)	<0.0001	181 (54.4%)	0.036	1015 (45.9%)	0.018	78 (32.3%)	<0.0001
**Neuropathy**	**Yes**	10387 (19.1%)	7928 (16.3%)	2459 (42.4%)	0.001	94 (24%)	<0.0001	1244 (48.3%)	<0.0001	169 (36.7%)	<0.0001
**Retinopathy**	**Yes**	9749 (19.4%)	7011 (15.7%)	2738 (48.8%)	<0.0001	91 (23.8%)	<0.0001	1518 (60.1%)	<0.0001	188 (41%)	<0.0001
**Vasculopathy**	**Yes**	10110 (18.5%)	7972 (16.4%)	2138 (36.6%)	<0.0001	55 (13.9%)	0.169	756 (29.2%)	<0.0001	244 (50.4%)	<0.0001
**Hypertension**	**Yes**	28026 (51.4%)	23541 (48.4%)	4485 (76.2%)	<0.0001	275 (69.4%)	<0.0001	1821 (70.7%)	<0.0001	443 (91.7%)	<0.0001
**Hyperlipidemia**	**Yes**	20789 (38.2%)	17989 (37.0%)	2800 (48.0%)	<0.0001	264 (66.7%)	<0.0001	1077 (42.1%)	<0.0001	189 (40.9%)	0.084
**Insulin therapy**	**Yes**	6098 (11.2%)	4983 (10.2%)	1115 (18.9%)	<0.0001	27 (6.8%)	0.025	504 (19.5%)	<0.0001	78 (16.1%)	<0.0001
**HbA1c>8%**	**Yes**	2,253 (15.1%)	1,167(9.3%)	935 (60.6%)	<0.0001	132 (61.1%)	<0.0001	284 (59.5%)	<0.0001	35 (63.6%)	0.0001

*P value is the difference between non-nephropathic patients and microabluminureia, macroalbuminuria, and ESRD patients. Frequency analysis was adjusted according to the data availability.

The highest frequency was seen among the 45 to 64 age group in the total sample (52.2%), among non-nephropathic (53.3%) and microalbuminuria (59.9%) patients, but among age group ≥65 years, in total nephropathic (51.2%), macroalbuminuria (53.2%) and ESRD (52.3%) patients.

Males had higher frequency in the total sample or nephropathic patients, regardless of the nephropathy types, but the highest in macroalbuminuria and ESRD, with a percentage of 60.2% and 64.3% respectively. When comparing the marital status of nephropathic patients with their non nephropathic counterparts, there was a significantly lower percentage of singles, but a higher percentage of widows among nephropathic patients (2.1% vs 1.5% and 5.2% vs 6.5%), (p = 0.001, <0.0001). The presence of diabetes family history was significantly higher among nephropathic and patients with microalbuminuria and macroalbuminuria, (p<0.0001), but not with ESRD patients, (p = 0.866), represented by 43.9%, 49.4%, 45.1%, 36.5% respectively. Smoking was significantly more prevalent among nephropathy patients in general, at 10.9%, and at 13.9% for ESRD patients, (p = 0.001, 0.029) respectively, but not in patients with microalbuminuria or macroalbuminuria.

Extremes of BMI of ≤25 and ≥30 kg/m^2^ had significant differences in the total nephropathic patients, when compared with non-nephropathic patients. The percentage was higher in patients with microalbuminuria in ≥30 kg\m^2^ BMI patients at 54.4%, and was significantly higher in the two extremes of BMI in macroalbuminuria at 20% for BMI ≤25 and 45.9% for BMI ≥30. This was the same for ESRD patients, where 35.7% for BMI ≤25 and 32.3% for BMI ≥30.

Prevalence of neuropathy was significantly higher in nephropathic patients as a whole, accounting for 42.4% and each type, namely microalbuminuria, macroalbuminuria, and ESRD 24%, 48.3%, and 36.7%, (p = 0.001, <0.0001, <0.0001, <0.0001) respectively. Retinopathy prevalence was significantly higher among nephropathic patients 48.8% and 23.8%, 60.1% and 41% for microalbuminuria, macroalbuminuria, and ESRD respectively, (p<0.0001).

Vasculopathy as ischemic heart disease, cerebrovascular disease or periphrovascular disease was found in 36.6% of nephropathic patients, and was significantly higher in macroalbuminuria, (p<0.0001) and ESRD but not in microalbuminuria. Hypertension prevalence was significantly higher among microalbuminuria, macroalbuminuria and ESRD patients, accounting for 69.4%, 70.7% and 91.7%, (p<0.0001) respectively, with an overall prevalence of 76.2%. Hyperlipidemia prevalence was 48.0% in patients with nephropathy, and significantly higher in microalbuminuria and macroalbuminuria patients 66.75 and 42.1%, (p<0.0001) respectively, but not in patients with an ESRD of 40.9%. 18.9% of patients with nephropathy were using insulin, which is significantly higher than non-nephropathic patients, (p<0.0001). This was true for macroalbuminuria, and ESRD 19.5%, and 16.1%, but significantly lower in patients with microalbuminuria 6.8%. Poor glycemic control, represented by the percentage of patients with HbA1c >8%, showed a significantly higher percentages in nephropathic patients, microalbuninuria, macroalbuminuria and ESRD, representing 60.6%, 61.1%, 59.5% and 63.6% respectively with p value <0.0001.

**Figure 2:**
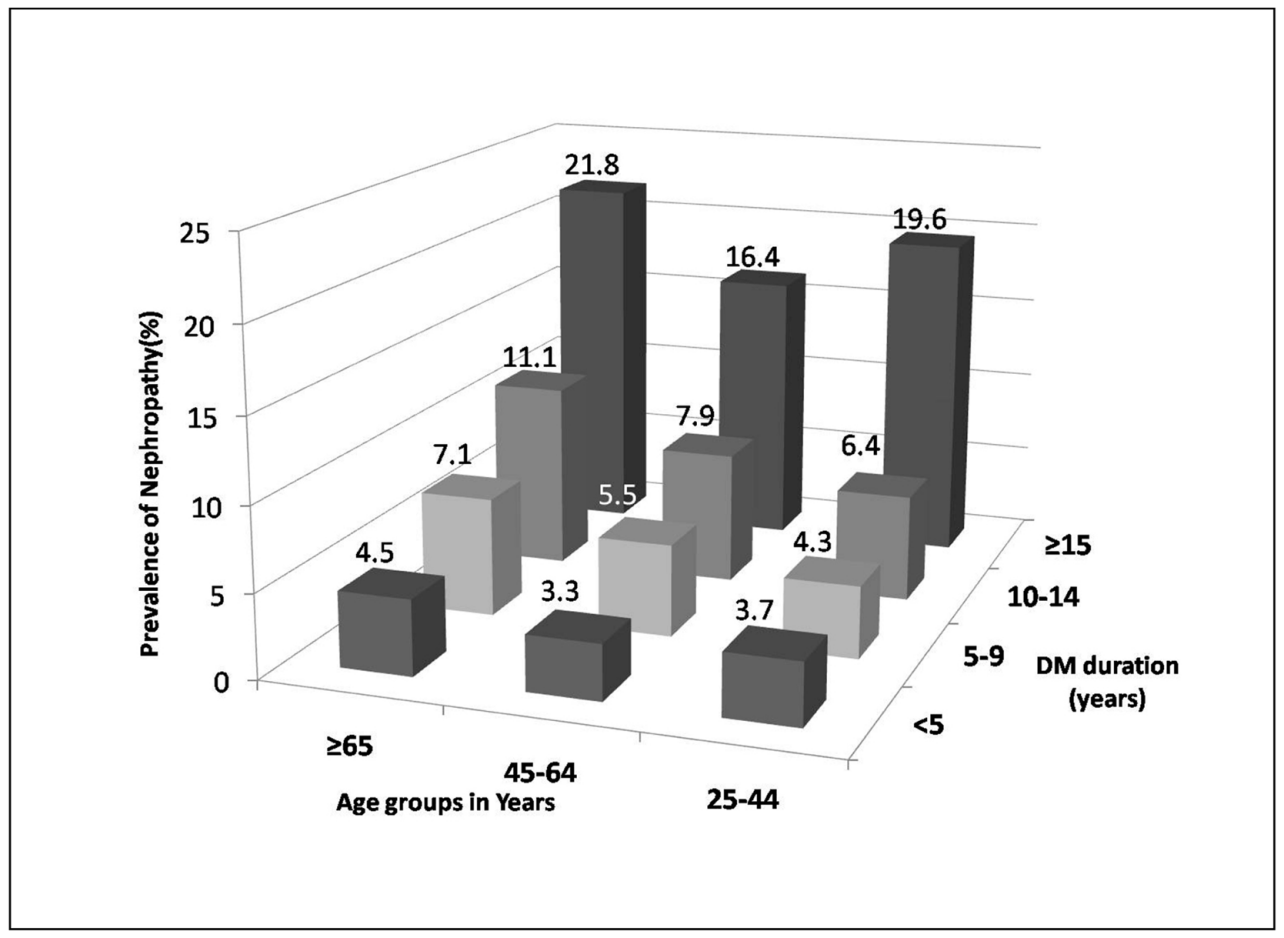
Three dimensional figure for prevalence of diabetic nephropathy according to age and diabetes duration grouping. Nephropathy prevalence calculated per group according to age in relation to the five years duration for the total of 54,670 patients.

Figure 2 shows the correlation between patients' age groups and diabetes duration in relation to the prevalence of diabetic nephropathy. The prevalence of nephropathy multiplied with increase in age and diabetes duration. The lowest prevalence was in 25–44 and 45–64 years age groups, with diabetes duration <5 years represented by 3.7% and 3.3% respectively, while the highest was with duration ≥15 years in age groups 25–44, 45–64 and ≥65 years, amounting to 19.6%, 16.4% and 21.8%.

**Figure 3:**
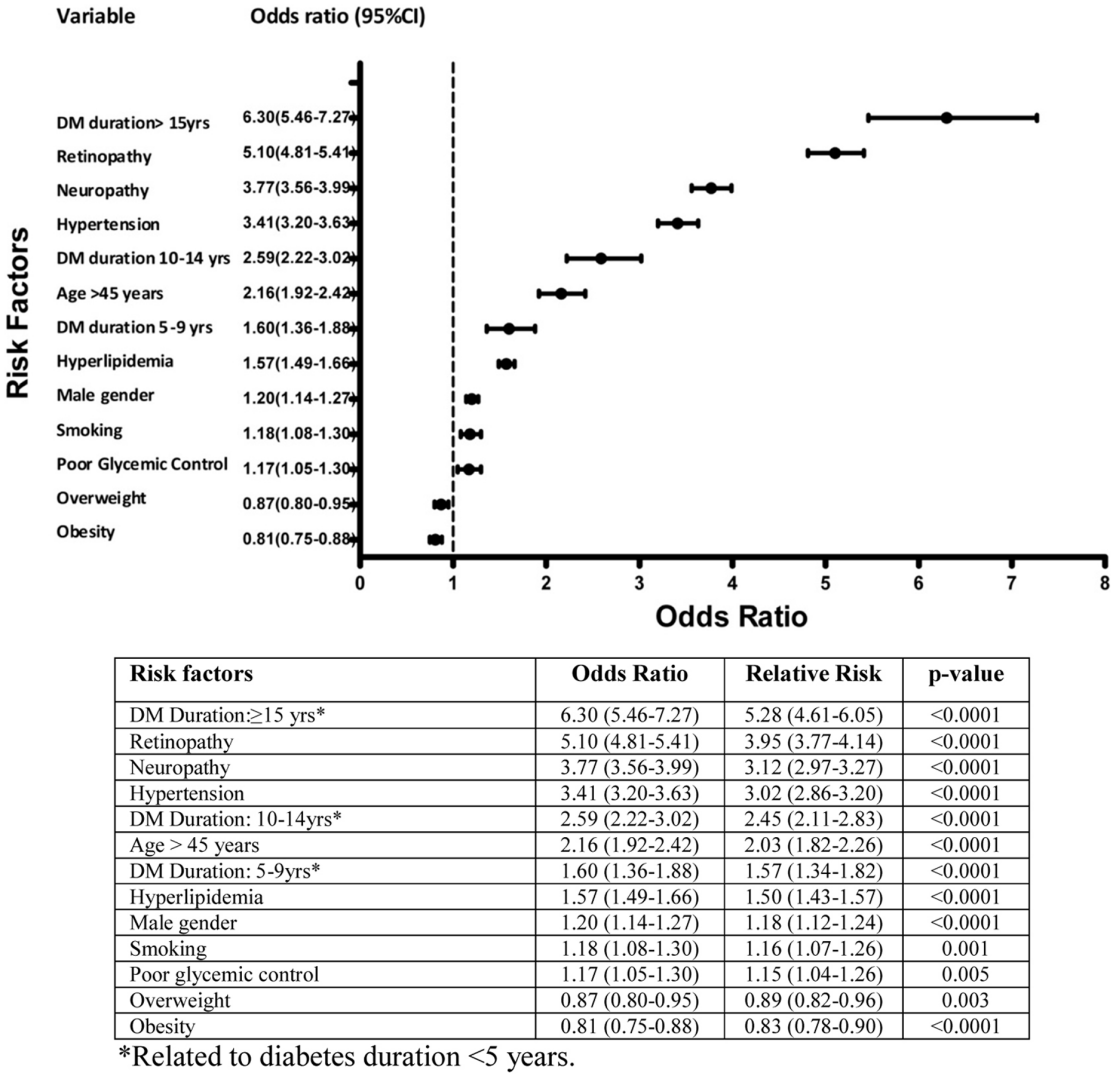
Forest plot for odds ratio & 95% confidence interval for diabetic nephropathy risk. * Related to diabetes duration <5 years.

Figure 3 is the forest plot for odds ratio (OR) and relative (RR) risk for diabetic nephropathy risk factors in the studied sample. Diabetes duration was the most significant risk with OR (95%CI) being 6.30 (5.46–7.27), 2.59 (2.22–3.02) and 1.60 (1.36–1.88) for the duration ≥15, 10–14, and 5–9 years. Retinopathy and neuropathy were the second and third significant risk factors with OR (95%CI) of 5.10 (4.81–5.41) and 3.77 (3.56–3.99) respectively. Patients aged >45 years had an OR (95%CI) of 2.16 (1.92–2.42) followed by hyperlipidemia, male gender, and smoking, with OR (95%CI) 1.57 (1.49–1.66), 1.20(1.14–1.27) and 1.18 (1.08–1.30) respectively. Poor glycemic control had the lowest but significant OR (95%CI) of 1.17 (1.05–1.30). Overweight and obesity showed significant protective risk for diabetic nephropathy, with OR (95%CI) 0.87 (0.80–0.95) and 0.81(0.75–0.88) respectively.

Table 3 demonstrates OR and RR for risk factors related to the different types of diabetic nephropathy. In patients with microalbumiuria OR (95% CI) was the highest, with hyperlipdemia mounting for 3.4 (2.76–4.20) followed by hypertension mounting for 2.42 (2.0–3.0). Retinopathy and neuropathy had OR of 1.67 (1.32–2.11) and 1.62 (1.29–2.05) respectively. Obesity had a significant OR of 1.43 (1.03–1.98), but diabetes duration did not show any significant risk for microlabuminuria, which was also true for age >45 years, male gender, smoking, poor glycemic control, and overweight. Significant risk factors seen with macroalbuminuria were diabetes duration ≥15 years with OR (95% CI) of 8.00 (6.38–10.05) and presence of retinopathy followed by neuropathy with OR (95% CI) of 8.07 (7.43–8.78) and 4.48 (4.42–5.20). Hypertension, age >45 years, and hyperlipedemia had OR (95% CI) of 2.57 (2.36–2.80), 2.52 (2.10–3.03) and 1.24 (1.14–1.34) respectively for macroalbuminuria.While male gender showed a significantly low OR (95% CI) of 0.73 (0.68–0.79). Microalbuminuria and macroalbuminuria had no significant OR or RR for smoking, poor glycemic control and overweight, but this was not the case for obesity, which had an increased OR (95% CI) and RR (95% CI) of 1.43 (1.03–1.98) and 1.43 (1.03–1.97) respectively for microalbuminuria and a decreased OR (95% CI) of 0.84 (0.75–0.94) and RR of 0.85 (0.76–0.94). Both overweight and obesity had significant reduced risk for ESRD, while smoking had a significant increased risk with OR (95% CI) and RR (95% CI) of 1.41 (1.03–1.92) and 1.41 (1.03–1.92), but non-significant increase for poor glycemic control.

**Table 3:** Multiple logistic regression analysis of risk factors for different type of nephropathy.

Risk factors	Microalbuminuria			Macroalbuminuria			ESRD		
	OR (95%CI)	RR (95%CI)	p-value	OR (95%CI)	RR (95%CI)	p-value	OR (95%CI)	RR (95%CI)	p-value
**Duration** **≥15 years** *	1.38(0.97–1.96)	1.37(0.97–1.95)	0.077	8.00(6.38–10.05)	7.28(5.82–9.10)	<0.0001	12.77(6.32–25.81)	12.56(6.22–25.34)	<0.0001
**Retinopathy**	1.67(1.32–2.11)	1.66(1.31–2.10)	<0.0001	8.07(7.43–8.78)	6.81(6.31–7.35)	<0.0001	3.73(3.09–4.50)	3.65(3.04–4.39)	<0.0001
**Neuropathy**	1.62(1.29–2.05)	1.62(1.28–2.04)	<0.0001	4.48(4.42–5.20)	4.28(3.98–4.61)	<0.0001	2.97(2.45–3.60)	2.93(2.43–3.54)	<0.0001
**Hypertension**	2.42(2.0–3.0)	2.4(1.94–2.98)	<0.0001	2.57(2.36–2.80)	2.46(2.26–2.67)	<0.0001	11.81(8.54–16.32)	11.61(8.40–16.03)	<0.0001
**Duration** **10**–**14 years** *	1.22(0.84–1.79)	1.22(0.84–1.78)	0.301	3.05(2.40–3.89)	2.96(2.34–3.76)	<0.0001	4.22(2.02–8.83)	4.20(2.01–8.78)	<0.0001
**Age >45 years**	0.89(0.66–1.20)	0.89(0.66–1.20)	0.455	2.52(2.10–3.03)	2.44(2.04–2.91)	<0.0001	2.77(1.79–4.29)	2.75(1.78–4.26)	<0.0001
**Duration** **5**–**9 years** *	1.27(0.87–1.84)	1.26(0.87–1.84)	0.216	1.51(1.16–1.95)	1.50(1.16–1.93)	0.002	3.06(1.44–6.48)	3.05(1.44–6.45)	0.002
**Hyperlipidemia**	3.4(2.76–4.20)	3.37(2.74–4.15)	<0.0001	1.24(1.14–1.34)	1.22(1.13–1.32)	<0.0001	1.17(0.97–1.41)	1.10(0.99–1.23)	0.095
**Male gender**	0.88(0.72–1.08)	0.88(0.73–1.07)	0.213	0.73(0.68–0.79)	0.75(0.70–0.80)	<0.0001	1.47(1.22–1.77)	1.47(1.22–1.76)	<0.0001
**Smoking**	0.91(0.61–1.36)	0.91(0.61–1.35)	0.645	1.02(0.89–1.18)	1.02(0.90–1.17)	0.734	1.41(1.03–1.92)	1.41(1.03–1.91)	0.029
**Poor glycemic control**	1.19(0.90–1.57)	1.19(0.91–1.56)	0.214	1.12(0.93–1.34)	1.11(0.93–1.33)	0.251	1.33(0.77–2.30)	1.33(0.77–2.29)	0.313
**Overweight**	1.21(0.85–1.71)	1.20(0.85–1.70)	0.292	0.89(0.79–1.01)	0.90(80–1.01)	0.066	0.47(0.34–0.64)	0.47(0.35–0.64)	<0.0001
**Obesity**	1.43(1.03–1.98)	1.43(1.03–1.97)	0.03	0.84(0.75–0.94)	0.85(0.76–0.94)	0.003	0.33(0.24–0.45)	0.33(0.25–0.45)	<0.0001

*Related to diabetes duration <5 years.

In patients with ESRD, diabetes duration of ≥15 years had the highest significant OR (95% CI) of 12.77 (6.32–25.81), followed by the presence of hypertension with OR (95% CI) of 11.81 (8.54–16.32). Retinopathy, neuropathy and age >45 years were significant risk factors with OR (95% CI) of 3.73 (3.09–4.50), 2.97 (2.45–3.60) and 2.77 (1.79–4.29) respectively. Male gender and Smoking were the least risk factors, although still significant, with an OR (95% CI) of 1.47 (1.22–1.77) and 1.41(1.03–1.92). Poor glycemic control was not a significant risk factor, while both overweight and obesity had significant low risk in ESRD with OR (95% CI) of 0.47 (0.34–0.64) and 0.33 (0.24–0.45) respectively.

## Discussion

The study sample used in this study represents the known age and gender distribution seen in Saudi society [5]. The overall prevalence of diabetic nephropathy among type 2 diabetic patients older than 25 years in the SNDR was 10.8%, which is similar to what has been reported by the UK primary care initiative 11.5% [7]. A similar cohort of type 2 diabetic patients reported by Mohan et al. [12] from India in the year 2000 showed a macroalbuminuria prevalence of 6.9%, which is less than reported in this study, and microalbuminuria of 2.5%, which is more than reported here. These findings are less than expected, as proven by different studies from the United States and India in the years 1993 and 2004, where microalbuminuria and macroalbuminuria were reported to be (25.9% and 32.1%) and (17.6% and 2.2%) respectively [13], [14].

This is true in the Saudi cross sectional hospital based study, where microalbuminuria was reported to be 41.3% in 1994 [15]. The low percentage of microalbuminuria and macroalbuminuria reported by SNDR patients resulted from a lack of screening programs in most health institutions, which is not the case in ESRD cases that do not need screening, but usually discovered because of the acute presentation and clinical symptoms.

Saudi men with type 2 diabetes have a higher prevalence of diabetes nephropathy, as has been observed by similar studies in different communities [16], [17]. This may be explained by the fact that the estrogen hormone plays an important role in protection [18].

In this study, men have a higher risk of ESRD, which differs from what has been found by the Denmark and Korean studies. This may be explained by the lower number of male patients [16], [19].

The prevalence of ESRD in this cohort is three times that reported by the Thailand Diabetes Registry, where it was 0.47% [8]. This may be explained by the high percentage of poor glycemic control, retinopathy and hypertension, in addition to the longer duration of diabetes among the SNDR cohort. This study reports on the age effects of both microalbuminuria and macroalbuminuria, which are similar to the CURES study in India [14].

In comparing this registry with similar registries, BMI was found to be higher among Saudi diabetic nephropathy patients than has been reported in Danish people [16], due to the high prevalence of obesity and being overweight among the Saudi population [20].

There is a strong correlation between age and diabetes duration, as seen in other published data [14], [21]. We report for the first time the accumulative effect of both age and duration on the prevalence of diabetic nephropathy, where it increases by about five times in patients with diabetes duration ≥15 years, regardless of the patient’s age.

The forest plot shows diabetes duration to be the most important risk factor, especially ≥15 years. This was the same finding in different ethnic groups, as shown in Korean, Indian and Taiwanese studies [19], [21], [6]. In the Saudi population, the hazard ratio for diabetes nephropathy was found to be 2.3 with a duration>10 years [11]. The role of diabetes duration has been proven by the UKPDS, where approximately one quarter of patients, developed microalbuminuria or worsening nephropathy after ten years [22].

Diabetic retinopathy is a known risk factor for diabetic nephropathy in different ethnic populations, as reported in Taiwanese and Danish studies [6], [16]. Since diabetic neuropathy has been found by the UKPDS to be a risk factor for renal insufficiency or mircoalbuminuria and macrolbuminuria [23], this study move in the same direction, proving that neuropathy has a higher OR and RR. Hypertension presents a significant risk for nephropathy in general, and with each type of diabetic nephropathy, which was also observed by another Saudi study [11]. Age alone has been found to be an important risk factor, which is the same observation in this study, but not for microalbuminuria patients [16], [17].

The RENAAL study showed that dyslipidemia was associated with greater hazards in the development of a renal end point [24], and high cholesterol, LDL and triglyceride has been proven to be a risk for diabetic nephropathy by many studies [17], [23]. This is also true in this study, with significantly increased OR and RR when looking at hyperlipidemic patients, but was not significant for ESRD cases. Male gender is a known risk factor in many studies, which is true in this study but not for microalbuminuric subjects, which could be explained by underestimation due to underscreening [16], [17]. This study has a lower OR and RR for smoking than reported by other studies [17], [19], [25] due to low smoking prevalence among Saudi women [26].

The role of strict control on the progression of diabetic nephropathy is not firmly established [27], although poor glycemic control is a recognized cause of diabetic nephropathy [6], [27], [28]. In agreement with a number of previous studies, we also demonstrate that poor glycemic control is associated with diabetic nephropathy [29].

We have identified being overweight or obesity as a strong and potentially modifiable risk factor for the development of ESRD, which was the same observation as seen in other studies [30].

Our observation shows that Obesity seems to be an important -and potentially preventable- risk factor for ESRD that may be explained by weight loss among case patients, as a consequence of morbidity related to renal failure itself, as also seen by another study [31].

In conclusion, diabetic nephropathy is underestimated in the SNDR as being due to lack of screening programs, an observation that is shared by other registries. The current data confirms that the most significant risk factors for diabetic nephropathy in Saudi type 2 diabetic population are diabetes duration, retinopathy, neuropathy, hypertension, age >45 years, hyperlipidemia, smoking, and poor glycemic control chronologically. Risk factors for microalbuminuria and macroalbuminuria are retinopathy, neuropathy, hypertension, hyperlipidemia and obesity, while diabetes duration, age >45 years and male gender are risk factors for macroalbuminuria only. ESRD risk factors are the same except for hyperlipidemia and poor glycemic control. There is an urgent need to launch a screening program for diabetic nephropathy, and to start prevention to protect the kidney in diabetic patients.

## References

[1] Carstensen B, Kristensen JK, Ottosen P, BorchJohnsen K (2008) Steering Group of the National Diabetes Register (2008) The Danish National Diabetes Register: Trends In Incidence, Prevalence And Mortality. Diabetologia 51(12): 2187–96. 18815769 10.1007/s00125-008-1156-z

[2] Ford DJ, Fogarty DG, Steenkamp R, Tomson CRV, Ben-Shlomo Y, et al (2010) Chapter 13: The UK Renal Registry Advanced CKD Study: frequency of incorrect reporting of date of start of RRT. Nephron Clinical Practice 115(1): c271–c78. 20413951 10.1159/000301236

[3] Patient Registries (2010) In: Gliklich RE, Dreyer NA, (eds) Registries for Evaluating Patient Outcomes: A User's Guide, 2nd edition. Agency for Healthcare Research and Quality (US) http://www.ncbi.nlm.nih.gov/books/NBK49448/. 21204321

[4] Middle East and North Africa (MENA) Diabetes Atlas (2011), 5^th^ ed. http://www.idf.org/diabetesatlas/5e/middle-east-and-north-africa.

[5] AlNozha MM, AlMaatouq MA, AlMazrou YY, AlHarthi SS, Arafah MR, et al (2004) Diabetes mellitus In Saudi Arabia. Saudi Med J 25(11): 1603–10. 15573186

[6] Shen FC, Chen CY, Su SC, Liu RT (2009) The Prevalence And Risk Factors Of Diabetic Nephropathy In Taiwanese Type 2 Diabetes - A Hospital Based Study. ActaNephrologica 23: 2.

[7] Magee GM, Hunter SJ, Cardwell CR, Savage G, Kee F, et al (2010) Identifying additional patients with diabetic nephropathy using the UK primary care initiative. Diabet Med 27(12): 1372–8 10.1111/j.14645491.2010.03105.x 21059089

[8] Ngarmukos C, Bunnag P, Kosachunhanun N, Krittiyawong S, Leelawatana R, et al (2006) Thailand diabetes registry project: prevalence, characteristics and treatment of patients with diabetic nephropathy. J Med Assoc Thai 89 (1): S37–42. 17715832

[9] Alwakeel JS, Sulimani R, AlAsaad H, AlHarbi A, Tarif N, et al (2008) Diabetes complications in 1952 type 2 diabetes mellitus patients managed in a single institution in Saudi Arabia. Ann Saudi Med 28(4): 260–6. 18596402 10.5144/0256-4947.2008.260 PMC6074352

[10] Raile K, Galler A, Hofer S, Herbst A, Dunstheimer D, et al. (2007) Diabetic Nephropathy in 27,805 Children, Adolescents, and Adults With Type 1 Diabetes. Diabetes Care 30(10): 2523-8. 10.2337/dc07-0282 17630266

[11] Alwakeel JS, Isnani AC, Alsuwaida A, AlHarbi A, Shaikh SA, et al (2011) Factors affecting the progression of diabetic nephropathy and its complications: A single-center experience in Saudi Arabia. Ann Saudi Med 31(3): 236–242. 21623051 10.4103/0256-4947.81528 PMC3119962

[12] Mohan V, Meera R, Premalatha G, R Deepa, P Miranda, et al (2000) Frequency of proteinuria in type 2 diabetes mellitus seen at a diabetes centre in southern India. Postgrad Med J 67: 569–573. 10.1136/pmj.76.899.569 PMC1741744 10964123

[13] Klein R, Klein BE, Moss SE (1993) Prevalence of microalbuminuria in older-onset diabetes Diabetes Care. 16: 1325–1330. 10.2337/diacare.16.10.1325 8269789

[14] Unnikrishnan R, Rema M, Pradeepa R, Deepa M, Shanthirani CS, et al (2007) Prevalence And Risk Factors Of Diabetic Nephropathy In An Urban South Indian Population. Diabetes Care 30: 8. 17488949 10.2337/dc06-2554

[15] Alzaid AA, Sobki S, De Silva V (1994) Prevalence of microalbuminuria in Saudi Arabians with non-insulin-dependent diabetes mellitus: a clinic-based study. Diabetes Res Clin Pract 26(2): 115–20. 7705192 10.1016/0168-8227(94)90148-1

[16] Gall MA, Hougaard P, BorchJohnsen K, Parving HH (1997) Risk factors for development of incipient and overt diabetic nephropathy in patients with non-insulin dependent diabetes mellitus: prospective, observational study. BMJ 314(7083): 783–8. 9080995 10.1136/bmj.314.7083.783 PMC2126209

[17] Ravid M, Brosh D, RavidSafran D, Levy Z, Rachmani R (1997) Main risk factors for nephropathy in type 2 diabetes mellitus are plasma cholesterol levels, mean blood pressure, and hyperglycemia. Arch Intern Med 1998 158(9): 998–1004. 10.1001/archinte.158.9.998 9588433

[18] Maric C, Sullivan S (2008) Estrogens and the Diabetic Kidney. Gend Med 5 Suppl A: S103–13. doi:10.1016/j.genm.2008.03.010. PMC3178838 18395675

[19] Yang CW, Park KT, Kim YS, Kim YL, Lee YS (2011) Prevalence of diabetic nephropathy in primary care type 2 diabetic patients with hypertension: data from the Korean Epidemiology Study on Hypertension III (KEY III study). Nephrol Dial Transplant 26: 3249–3255. 21372264 10.1093/ndt/gfr011

[20] AlNozha MM, AlMazrou Y, AlMaatouq M, Arafah MR, Khalil MZ, et al (2005) Obesity in Saudi Arabia. Saudi Med J 26 (5): 824–829. 15951877

[21] Viswanathan V, Tilak P, Kumpatla S (2012) Risk factors associated with the development of overt nephropathy in type 2 diabetes patients: A 12 years observational study. Indian J Med Res 136: 46–53. 22885263 PMC3461717

[22] Adler AI, Stevens RJ, Manley SE, Bilous RW, Cull CA, et al (2003) Development and progression of nephropathy in type 2 diabetes: The United Kingdom Prospective Diabetes Study (UKPDS 64). Kidney International 63: 225–232 10.1046/j.1523-1755.2003.00712.x 12472787

[23] Retnakaran R, Cull CA, Thorne KI, Adler AI, Holman RR, et al. (2006) Risk Factors for Renal Dysfunction in Type 2 Diabetes U.K. Prospective Diabetes Study 74. Diabetes 55: 6 1832–1839. 10.2337/db05-1620 16731850

[24] Keane WF, Brenner BM, Zeeuw D, Grunfeld JP, McGill J, et al (2003) The risk of developing end-stage renal disease in patients with type 2 diabetes and nephropathy: The RENAAL Study. Kidney International 63: 1499–1507. 12631367 10.1046/j.1523-1755.2003.00885.x

[25] Mehler PS, Jeffers BW, Biggerstaff SL, Schrier RW (1998) Smoking as a Risk Factor for Nephropathy in Non-Insulin-Dependent Diabetics. J Gen Intern Med 13: 842–845. 9844083 10.1046/j.1525-1497.1998.00249.x PMC1497038

[26] Jarallah JS, AlRubeaan KA, AlNuaim AR, AlRuhaily AA, Kalantan KA (1999) Prevalence and determinants of smoking in three regions of Saudi Arabia. Tobacco Control 8: 53–56. 10465816 10.1136/tc.8.1.53 PMC1763927

[27] Gross JL, Azevedo MJ, Silveiro SP, Canani LH, Caramori ML, et al (2005) Diabetic Nephropathy: Diagnosis, Prevention, and Treatment. Diabetes Care 28: 176–188. 10.2337/diacare.28.1.164 15616252

[28] Fioretto P, Bruseghin M, Berto I, Gallina P, Manzato E, et al (2006) Renal protection in diabetes: role of glycemic control. J Am SocNephrol 4(2): S86–9. 10.1681/ASN.2005121343 16565255

[29] Bash LD, Selvin E, Steffes M, Coresh J, Astor BC (2008) Poor Glycemic Control in Diabetes and the Risk of Incident Chronic Kidney Disease Even in the Absence of Albuminuria and Retinopathy Atherosclerosis Risk in Communities (ARIC) Study. Arch Intern Med 168(22): 2440–2447 10.1001/archinte.168.22.2440 19064828 PMC2766035

[30] Hsu CY, McCulloch CE, Iribarren C, Darbinian J, Go AS (2006) Body Mass Index and Risk for End-Stage Renal Disease. Ann Intern Med 144: 21–28. 16389251 10.7326/0003-4819-144-1-200601030-00006

[31] Ejerblad E, Fored CM, Lindblad P, Fryzek J, McLaughlin JK, et al (2006) Obesity and Risk for Chronic Renal Failure J Am Soc Nephrol. 17: 1695–1702. 10.1681/ASN.2005060638 16641153

